# Bioinformatics and Structural Analysis of Antigenic Variation in the Hemagglutinin Gene of the Influenza A(H1N1)pdm09 Virus Circulating in Shiraz (2013 to 2015)

**DOI:** 10.1128/spectrum.04630-22

**Published:** 2023-07-12

**Authors:** Mojtaba Mortazavi, Neda Pirbonyeh, Fatemeh Javanmardi, Amir Emami

**Affiliations:** a Department of Biotechnology, Institute of Science and High Technology and Environmental Sciences, Graduate University of Advanced Technology, Kerman, Iran; b Microbiology Department, Burn and Wound Healing Research Center, Shiraz University of Medical Sciences, Shiraz, Iran; c Department of Bacteriology and Virology, Shiraz University of Medical Sciences, Shiraz, Iran; d Biostatistics Department, Shiraz Medical School, Shiraz University of Medical Sciences, Shiraz, Iran; Sechenov Institute of Evolutionary Physiology and Biochemistry, RAS

**Keywords:** antigenic variation, hemagglutinin, influenza A, H1N1

## Abstract

Circulating influenza A virus provided an excellent opportunity to study the adaptation of the influenza A(H1N1)pdm09 virus to the human host. Particularly, due to the availability of sequences taken from isolates, we could monitor amino acid changes and the stability of mutations that occurred in hemagglutinin (HA). HA is crucial to viral infection because it binds to ciliated cell receptors and mediates the fusion of cells and viral membranes; because antibodies that bind to HA may block virus entry to the cell, this protein is subjected to high selective pressure. In this study, the locations of mutations in the structures of mutant HA were analyzed and the three-dimensional (3D) structures of these mutations were modeled in I-TASSER. Also, the location of these mutations was visualized and studied using Swiss PDB Viewer software and the PyMOL Molecular Graphics System. The crystal structure of the HA from A/California/07/2009 (3LZG) was used for further analysis. The new noncovalent bond formations in mutant luciferases were analyzed via WHAT IF and PIC, and protein stability was evaluated in the iStable server. We identified 33 and 23 mutations in A/Shiraz/106/2015 and A/California/07/2009 isolates, respectively; some mutations are located on the antigenic sites of Sa, Sb, Ca1, Ca2, and Cb HA1 and the fusion peptide of HA2. The results show that with the mutation some interactions are lost and new interactions are formed with other amino acids. The results of the free-energy analysis suggested that these new interactions have a destabilizing effect, which needs confirmation experimentally.

**IMPORTANCE** Due to the fact that the mutations that occurred in the influenza virus HA cause the instability of the protein produced by the virus and antigenic changes and the escape of the virus from the immune system, the mutations that occurred in A/Shiraz/1/2013 were investigated in terms of energy level and stability. The mutations located in a globular portion of the HA are S188T, Q191H, S270P, K285Q, and P299L. On the other hand, the E374K, E46K-B, S124N-B, and I321V mutations are located in the stem portion of the HA (HA2). The change V252L mutation eliminates interactions with Ala181, Phe147, Leu151, and Trp153 and forms new interactions with Gly195, Asn264, Phe161, Met244, Tyr246, Leu165, and Trp167 which can change the stability of the HA structure. The K166Q mutation, which is located within the antigenic site Sa, causes the virus to escape from the immune response.

## INTRODUCTION

Influenza is a contagious viral infection that can cause mild to severe symptoms and life-threatening complications, including death, even in healthy children and adults (1). Influenza is caused by the *Orthomyxoviridae*, characterized by a negative-sense RNA that encodes 11 proteins (2). Influenza viruses are categorized as types A, B, C, and D. These major types generally produce similar symptoms, but their antigenic symptoms are not related to each other. Hence, infection with one type confers no immunity against the others (3). The influenza type A viruses cause great influenza epidemics, the influenza type B viruses cause smaller localized outbreaks, the influenza type C viruses cause only mild respiratory illness in humans, and the influenza type D viruses are not known to infect humans (4). Influenza A viruses are classified into subtypes that are differentiated mainly on the basis of two surface antigens, hemagglutinin (HA) and neuraminidase (NA). These proteins mediate host cell attachment and release. HA attaches virions to cells by binding to terminal sialic acid residues on glycoproteins/glycolipids to initiate the infectious cycle, while NA cleaves terminal sialic acids, releasing virions to complete the infectious cycle. These proteins are the primary targets of the protective antibody-mediated immune response (5). HA has functionally defined immunodominant antigenic sites that primarily map to the globular domain of the glycoprotein and surround the receptor binding site (RBS) (6). Circulation of influenza viruses gradually aggregates HA mutations (antigenic drift) or segment swapping (antigenic shift) in the antigenic sites targeted by neutralizing antibodies, allowing these mutations to escape the immune response and vaccination (5). Once that influenza virus acquires an HA protein through reconstruction, influenza pandemics may occur (7).

The influenza A(H1N1)pdm09 virus has been evolving since April 2009, acquiring new amino acid changes that may alter its antiviral drug susceptibility and antigenic and virulence characteristics (8). The protein structure of HA is composed of three monomers, each monomer composed of a heavy (HA1) (~40-kDa) and a light (HA2) (~20-kDa) chain that is held together by a disulfide bridge and noncovalent interactions (9). HA1 forms the receptor binding sites and antigenic sites (Sa, Sb, Ca1, Ca2, and Cb), while HA2 contains a conserved fusion peptide (10). These antigenic site residues are highly variable, and mutations are tolerated by viruses (11). Phylogenetic analysis of the HA gene of influenza A(H1N1)pdm09 viruses showed that it clustered into 8 genetic groups (12). Mutations in influenza virus HA proteins may alter the activity of influenza vaccines and antiviral drugs and are one of the possible catalysts for previous world pandemics (5). Many antiviral drug products, including vaccines, monoclonal antibodies (MAbs), and NA inhibitors (NAIs), target the HA or NA glycoproteins (5). Due to changes in the dynamic HA, the activity of these vaccines and drugs may be affected and the viral NA or HA protein mutations have reduced susceptibility to NAIs. For example, the D222G mutation in the HA1 subunit of HA was associated with severe clinical outcomes (12). These mutated viruses may be less transmissible but more pneumotropic and more resistant to antiviral treatment (13, 14). This resistance was generally associated with other mutations in the NA protein, although other mutations were also described to confer resistance to NAIs (15). Global virology monitoring needs to be updated for influenza virus mutations that may affect viral characteristics such as virulence, transmission, or antiviral susceptibility. NAI mutations in HA are likely to have the effect of lowering the receptor binding avidity and compensate for decreased activity of NA (16). However, it is not clear whether HA mutations are associated with decreased immune reactivity to anti-influenza virus antibodies.

Since the discovery of the first case of influenza A(H1N1)pdm09 in Fars province, Iran (15 July 2009), there have been many reports of influenza A(H1N1)pdm09 in patients with suspected influenza (14). As the HA mutations may affect the receptor binding specificity and strain pathogenicity, for the identification of emerging variants, continued epidemiological and molecular studies are essential for monitoring the modifications in the virus genome (17, 18). Also, evaluating the structural changes caused by these mutations in the protein is very important in understanding the danger level of the mutations and how the pathogenicity changes in this virus. Furthermore, the effects of these mutations on the function of the HA molecule were not studied. In this regard, we previously conducted a molecular and phylogenetic analysis of new influenza A/H1N1 (A/Shiraz/2013 and A/Shiraz/2015) virus strains that circulated in Fars province (19). In the present study, we investigated these identified HA mutations that may lead to changes in antigenic profiles and affect antibody-mediated virus inhibition. Amino acid sequence analysis of the HA gene (amino acids 1 to 566) indicated mutations in the HA1 domain, including Sa, Sb, Ca, and Cb (20), and in the HA2 region.

The HA genome shows the highest mutation rates that result in influenza virus genome instability (21). In this regard, the purpose of this study was to analyze the location of these identified mutations in the structure of HA. Furthermore, the three-dimensional (3D) structure of these mutated HAs was modeled in I-TASSER (20) and the location of these mutations was studied using Swiss PDB Viewer software (22) and the PyMOL Molecular Graphics System (23). The crystal structure of the HA from A/California/07/2009 (3LZG) (24) was used for further analysis. The new noncovalent bond formations in mutant luciferases were analyzed via WHAT IF (25) and PIC (26). In the following experiments, the effect of these mutations on protein stability was evaluated in the iStable server. These results help in elucidating the HA folding mechanism, as well as the rational design of new and effective drugs and diagnostic reagents. These data emphasize monitoring the process of influenza virus changes so that the vaccine composition can be changed according to the circulating strains.

## RESULTS AND DISCUSSION

### MSA.

**FIG 1:**
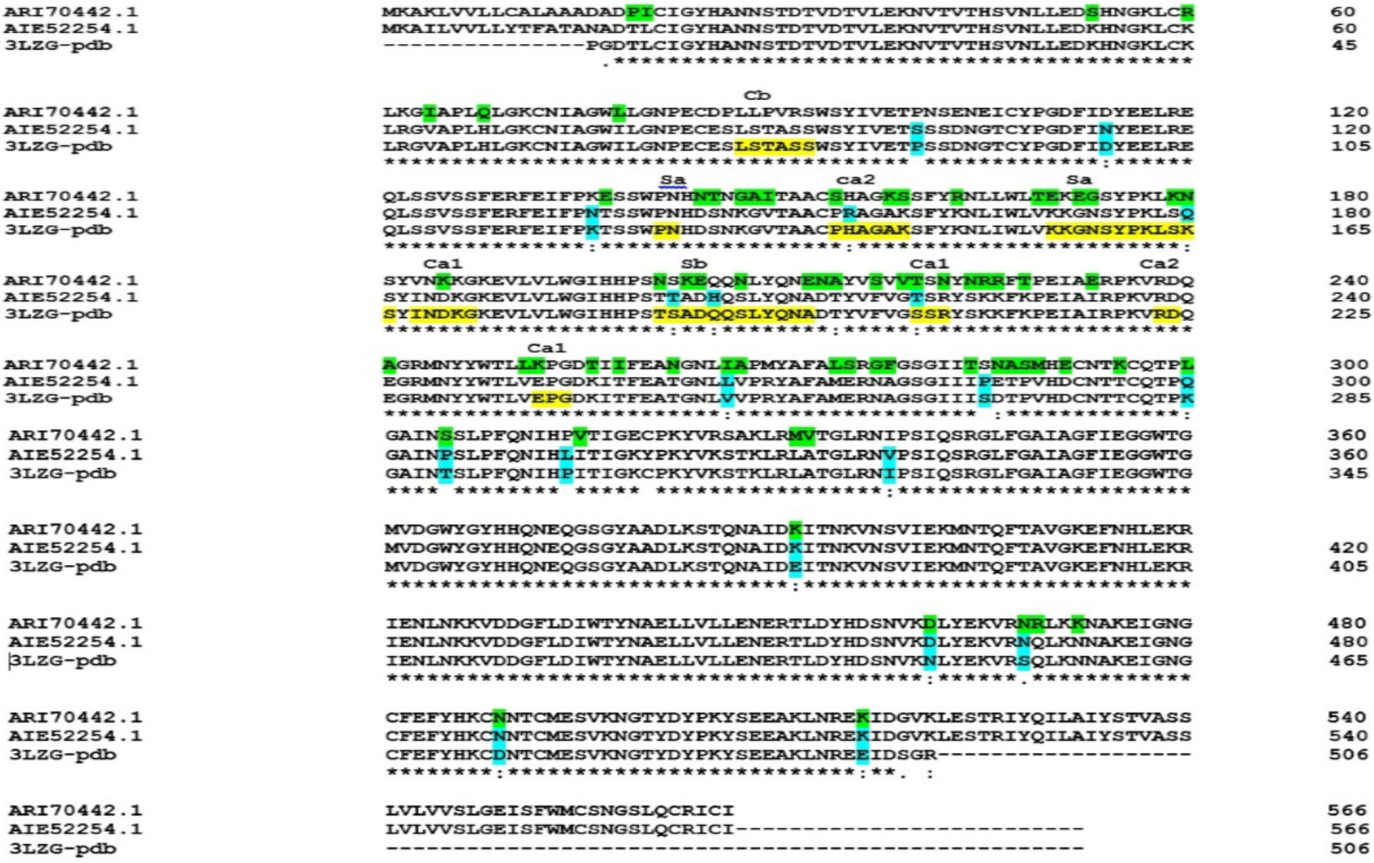
Multiple sequence alignment of HA sequences from A/Shiraz/1/2013 (AIE52254.1), A/Shiraz/106/2015 (ARI70442.1), and A/California/07/2009 (PDB: 3LZG) using Clustal Omega. The locations of the mutation in A/Shiraz/1/2013 and A/Shiraz/106/2015 are highlighted in blue and green, respectively. The amino acid sequence of antigenic sites for A/California/07/2009 virus is highlighted in yellow.

The amino acid sequence reference and mutated HA (ARI70442.1 and AIE52254.1, respectively) were retrieved and aligned in the Clustal Omega tool (https://www.ebi.ac.uk/Tools/msa/clustalo/). The multiple sequence alignment (MSA) was saved in the clustal_num format and analyzed with the Jalview software (Fig. 1 and data not shown). The crystal structure of the HA region from A/California/07/2009 (3LZG) was used for further analysis of the sequence alignment. The results show that some mutations compared to the A/California/07/2009 sample have been repeated in both the A/Shiraz/1/2013 (AIE52254.1) and A/Shiraz/106/2015 sequences. However, some mutations have occurred in only one of these. Also, the sequence of A/Shiraz/1/2013 (AIE52254.1) and A/Shiraz/106/2015 is about 45 nucleotides longer than the A/California/07/2009 sample (Fig. 1).

### Study of mutation effect on protein stability.

A relatively high number of mutations have been identified in the sequenced influenza A(H1N1)pdm09 virus that was collected from 2013 to April 2015 in Shiraz, Iran. The mutations are located in the HA1 (the globular portion of HA) and HA2 (stem portion of HA) domains of HA. The most common mutations were identified at the antigenic sites Ca, Sb, and Sa. Previous studies show that a single mutation by change of free energy (Δ*G*) may change the protein structural stability, while the difference in ΔΔ*G* between wild-type and mutant proteins is an impact factor in protein stability changes (27). In this regard, the effects of these mutations were evaluated in the iStable server. Free-energy analysis showed that most of these mutations have a slightly destabilizing effect (Table 1). It should be noted that in the iStable server, from the five web-based prediction tools that were chosen as element predictors, the final free-energy change was reported from the Meta result. While some of these element predictors may show positive results, the general conclusion reported is relevant to the Meta result that shows a single mutation by change of free energy may impact the protein structure stability. Some of these analyzed mutations are located in the stalk region. For universal influenza virus vaccines, the highly conserved HA stalk domain is an attractive target (28). In animal models antistalk antibodies are known to protect against a wide range of influenza viruses (29).

**TABLE 1:** Folding free-energy changes for selected mutations^*c*^

Mutation in PDB structure	Free-energy change (ΔΔ*G*, in kcal/mol)^*a*^
A/Shiraz/1/2013	
D104N	−0.627954
K123N	−0.997687
H141R	−0.209533
K166Q	−0.90291
S188T	−0.175269
Q191H	−0.53499
S206T	−0.606318
V252L	−0.88963
S270P	−0.678745
D271E	−0.43871
K285Q	−0.956947
T290P	−0.591243
P299L	−0.999348
C305Y	−0.0891661
I323V	−0.399771
E47K^*b*^	−0.512932
N117D^*b*^	−0.10797
S124N^*b*^	−0.163376
D145N^*b*^	−0.493296
E172K^*b*^	−0.043653
A/Shiraz/106/2015	
T12P	−0.722808
L13I	−0.843913
K46S	−0.472472
K53R	−0.697627
V56I	−0.866121
I70L	−0.314614
T124E	+0.269796
D131N	−1.46191
S132T	+0.48475
G134A	−1.2866
V135I	−0.10662
P140S	−1.34501
A144K	−0.66355
K145S	−0.401493
K149R	−0.604328
V155T	−0.977113
K156E	−0.5336
G158E	−0.755279
N159G	−0.583896
S165K	−0.797509
K166N	+0.881397
D171K	−0.474087
T187N	−0.635978
A189K	−0.511998
D190E	−0.263866
S193N	−0.301793
A198E	+0.25148
D199N	−0.0060
T200A	+1.08009
F203S	−1.94444
G205V	−1.18401
S206T	−0.606318
R208N	−0.884404
S210N	−0.56517
K211R	−0.561673
E227A	−0.988417
V237L	−0.735694
E238K	−1.30713
K212R	−0.391429
K214T	−0.181116
I219E	−0.929291
K242T	−0.928935
T244I	−0.878366
T248N	−0.663829
V252I	−0.484539
V253A	−1.51806
E261S	−0.708261
N263S	−1.02688
A264F	−0.477637
I269T	−1.29541
D271N	−1.03033
T272A	−1.10916
P273S	−1.44452
V274M	−0.924984
D276E	−0.332431
T289K	−0.836286
K285L	−0.507835
T290S	−0.440993
I300V	−0.615473
L316M	−0.470547
A317V	−0.469499
E47K^*b*^	−0.512932
N117D^*b*^	−1.10797
S124N^*b*^	−0.163376
Q125R^*b*^	−0.73738
N128K^*b*^	−1.14753
D145N^*b*^	−0.493296
E172K^*b*^	−0.043653

a Calculated with iStable. Positive values indicate a stabilizing effect, while negative values indicate a destabilizing effect.

b HA2 subunit.

c Abbreviations: ΔΔ*G*, free-energy change upon mutation; HA, hemagglutinin.

### Bioinformatics studies.

**FIG 2:**
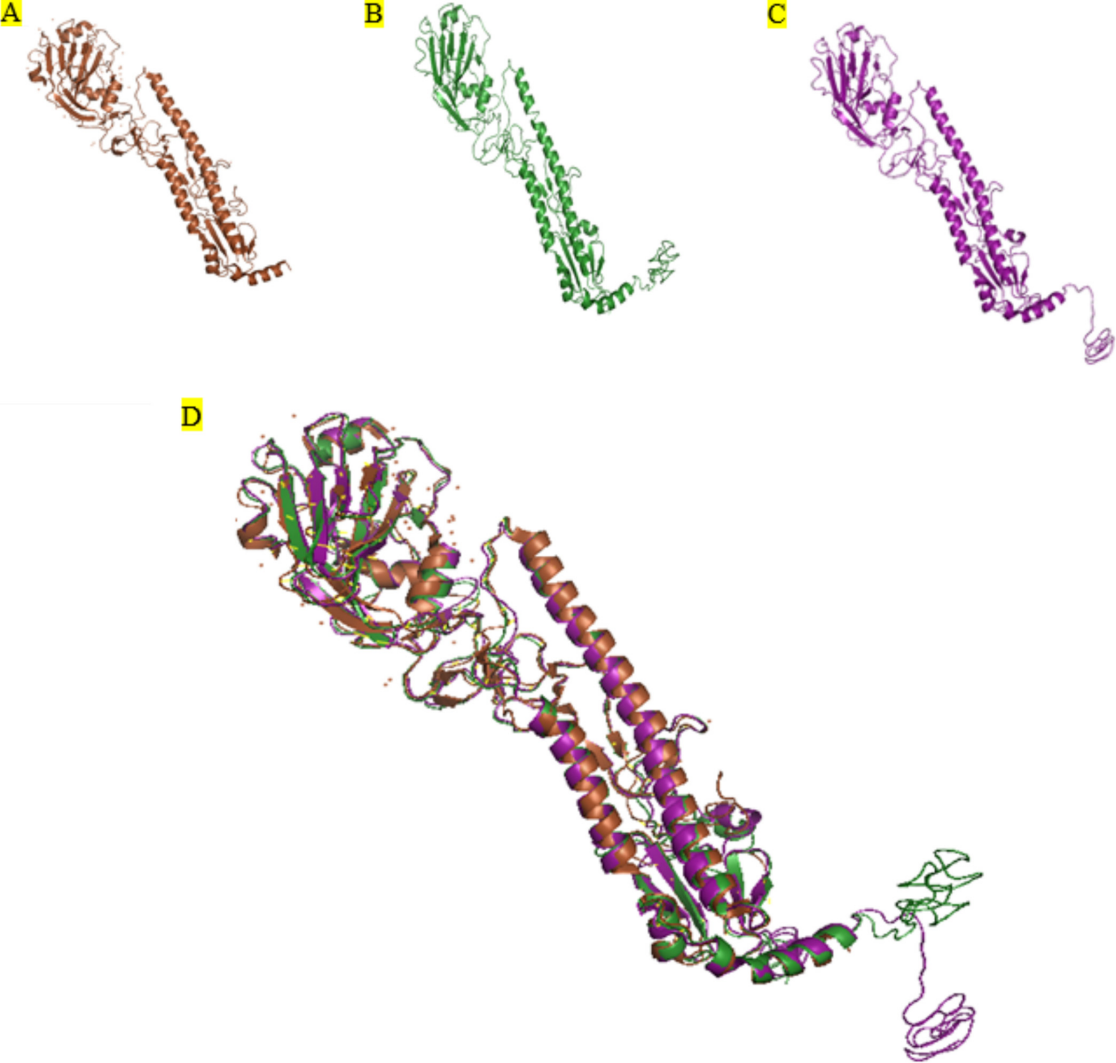
The ribbon diagram of HA (blue) from the crystal structure of the A/California/7/2009 H1N1 strain (brown) (PDB: 3LZG) (A), the 3D structure of the mutated HA from A/Shiraz/1/2013 (green) (B), the mutated A/Shiraz/106/2015 (magenta) (C), and superposition image of HA (A/California/7/2009 H1N1 strain) with mutated HA (HA from A/Shiraz/1/2013 and A/Shiraz/106/2015) (D).

For a better analysis of these mutations, the 3D structures of this HA were modeled in the I-TASSER server; the best model was selected (Fig. 2). These models of A/Shiraz/1/2013 and A/Shiraz/106/2015 show the 1.52 and 21.25 values of overall C-score, the 13.3 6 4.1 Å and 10.5 6 4.6 Å values of experimental root mean square deviations (RMSDs), and the 0.53 6 0.15 and 0.56 6 0.15 values of template modeling score (TM-score), respectively. For additional analysis we studied the locations of the mutated residues in the 3D structure of HA. Table 2 shows the physicochemical properties of HA from A/Shiraz/1/2013 that were calculated by the ProtParam tool. The results of this table show that the abundances of positively and negatively charged amino acids in the two proteins are similar. Instability index is a measure of proteins, used to determine the stability in a test tube. If the index is less than 40, then the protein is probably stable in the test tube. According to Table 2 the hemagglutinin of A/Shiraz/1/2013 is more stable than the hemagglutinin of A/Shiraz/106/2015.

**TABLE 2:** *In silico* physicochemical properties of HA from A/Shiraz/1/2013 and A/Shiraz/106/2015 obtained from ProtParam tool

No.	Parameter	Hemagglutinin from strain:	
		A/Shiraz/1/2013	A/Shiraz/106/2015
1	Theoretical pI	7.52	7.17
2	Mol wt	63,485.90	63,598.19
3	Sequence length	566	566
4	Extinction coefficient (M^−1^ cm^−1^ at 260 nm)^*a*^	96,720–97,595	93,740–94,740
5	Asp + Glu	59	61
6	Arg + Lys	60	61
7	Instability index	30.56	36.43
8	Grand avg of hydropathicity	−0.372	−0.371
9	Aliphatic index	81.98	83.37

a The first value is based on the assumption that both cysteine residues are oxidized and form cystine, and the second assumes that both cysteine residues are reduced. The number of Asp + Glu and Arg + Lys are obtained by counting the number of amino acids along the sequence.

Figures 2B and C show the three-dimensional models of HA from the influenza A/Shiraz/1/2013 H1N1 and A/Shiraz/106/2015 H1N1 viruses. These models were superimposed on the crystal structure of the 2009 H1N1 pandemic influenza virus (PDB: 3LZG) (Fig. 2A). The 3D structure analyses showed that the structures of these models have a high degree of similarity with the crystal structure of the 2009 H1N1 pandemic influenza virus (Fig. 2D).

**FIG 3:**
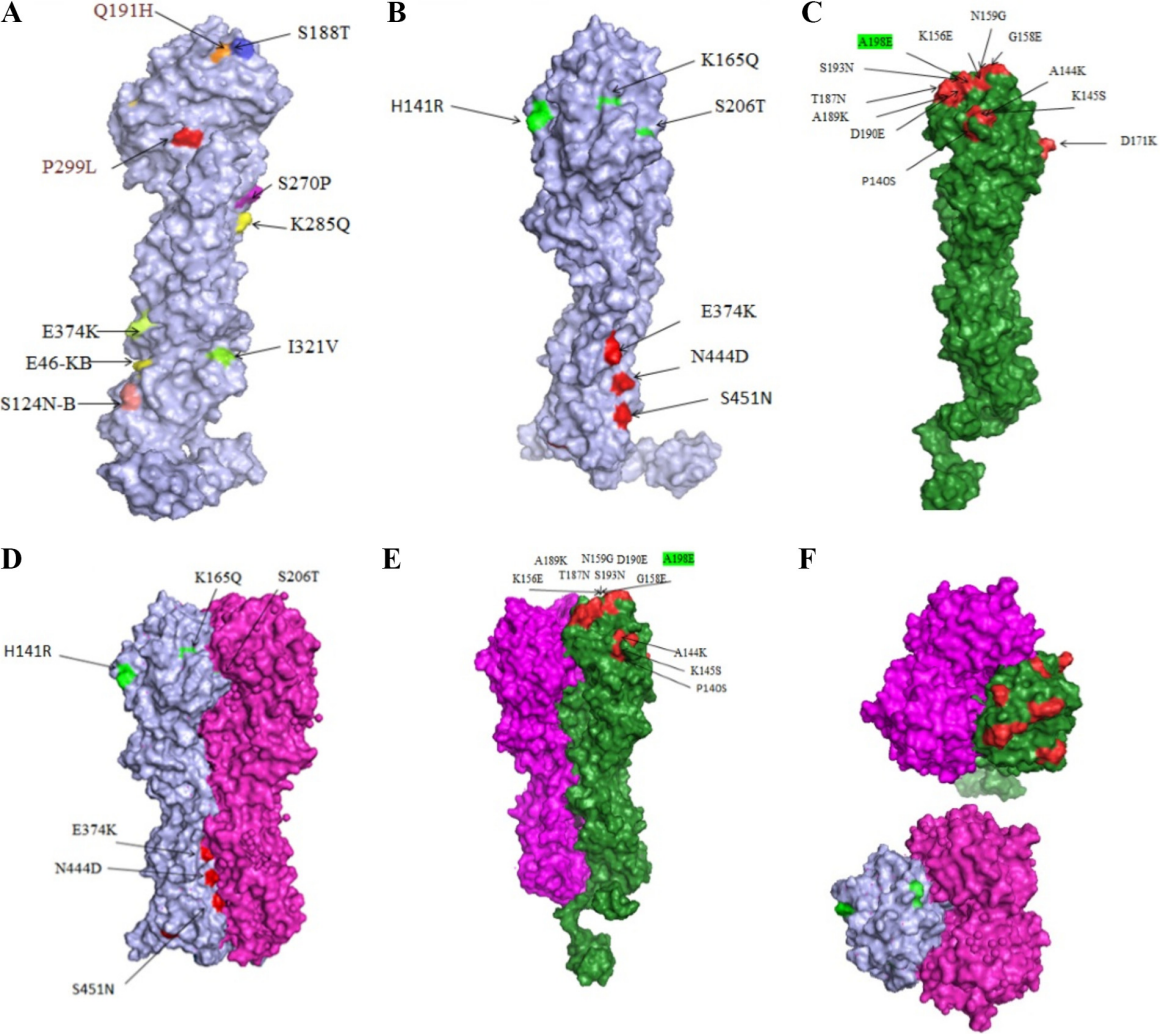
(A and B) Epitope mapping using HA point mutation in hemagglutinin from A/Shiraz/1/2013. (C) Epitope mapping using HA point mutation in hemagglutinin from A/Shiraz/106/2015. (D) Superimposition of hemagglutinin from A/Shiraz/1/2013 on the crystal structure of the HA from A/California/07/2009. (E) Superimposition of hemagglutinin from A/Shiraz/106/2015 on the crystal structure of the HA from A/California/07/2009. (F) View from the top of the model and the location of the mutation in relation to other subunits. Stereo presentation of the mutants’ location in the HA structure created by PyMOL. Sequence changes are shown and their locations are indicated on the 3D structure of HA. The structure is depicted as space-filling. Mutations are colored.

In the space-filling model of A/Shiraz/1/2013, the mutations located in HA1 (a globular portion of the HA) are S188T, Q191H, S270P, K285Q, and P299L (Fig. 3A, B, and D and Table 3). The E374K, E46K-B, S124N-B, and I321V mutations are located in HA2 (stem portion of the HA) (Fig. 3A). In the hemagglutinin of A/Shiraz/106/2015 the P140S, A144K, K145, SK156E, G158E, N159G, S165K, K166N, D171K, T187N, A189K, D190E, S193N, A198E, S206T, R208N, and E238K mutations are located in the HA1 domain (Fig. 3C and E and Table 3). In the space-filling model, other mutations were not visible due to their side chains not being surface exposed and being covered. This surface exposition of the side chain affects the mutation operation.

**TABLE 3:** Antigenic sites in structure of hemagglutinin

Mutation in antigenic site of hemagglutinin from virus	Antigenic site
A/Shiraz/1/2013	
D99N	
K123N	
H141R	Ca2
K166Q	Sa
S188T	Sb
Q191H	Sb
S206T	Ca1
V252L	
S268P	
D269E	
K285Q	
T288P	
P297L	
C305Y	
I321V	
E47K	Stalk
N117D	Stalk
S124N	Stalk
D145N	Stalk
E172K	
A/Shiraz/106/2015	
T12P	
L13I	
K46S	
K53R	
V56I	
I70L	
T124E	
D131N	
S132T	
G134A	
V135I	
P140S	Ca2
A144K	Ca2
K145S	Ca2
K149R	
V155T	
K156E	Sa
G158E	Sa
N159G	Sa
S165K	Sa
K166N	Sa
D171K	Ca1
T187N	Sb
A189K	Sb
D190E	Sb
S193N	Sb
A198E	Sb
D199N	
T200A	
F203S	
G205V	
S206T	Ca1
R208N	Ca1
S210N	
K211R	
K212R	
K214T	
I219E	
E227A	
V237L	
E238K	Ca1
K242T	
T244I	
T248N	
V252I	
V253A	
E261S	
N263S	
A264F	
I269T	
D271N	
T272A	
P273S	
V274M	
D276E	
T289K	
K285L	
T290S	
I300V	
L316M	
A317V	
E47K	Stalk
N117D	Stalk
S124N	Stalk
Q125R	Stalk
N128K	
D145N	Stalk
E172K	

**FIG 4:**
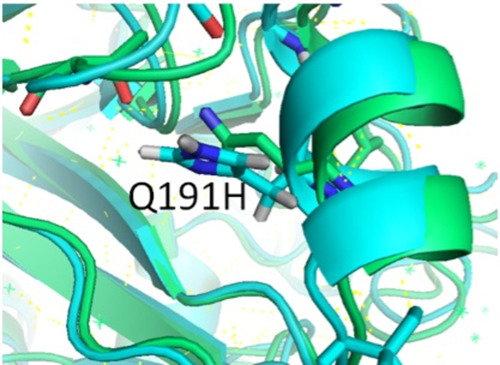
Stereo presentation of HA (hemagglutinin from A/Shiraz/1/2013) with location of Q191H created by PyMOL.

According to the previous results, a high number of mutations were identified as D97N, K119N, H138R, K163Q, S185T, Q191H, S203T, V252L, S268P, D269E, K285Q, T288P, P297L, C303Y, I321V, E374K, N444D, S451N, D472N, and E499K; we studied some of these mutations in the structure of hemagglutinin of A/Shiraz/1/2013. The 3D structure analysis showed that the Q191H mutation constitutes some interactions inside the HA structure (Fig. 4). The Q191H mutation is located in the globular part of HA and may have a role in the structural stability of HA (10) (Fig. 4). Mutation of Q191H may eliminate interactions with T200, N250, T187, S188, L194, Y195, and Q196 residues and forms a smaller number of interactions with His205, Ser207, Leu208, and Tyr209, which can reduce the stability of the HA structure (Table 3).

**FIG 5:**
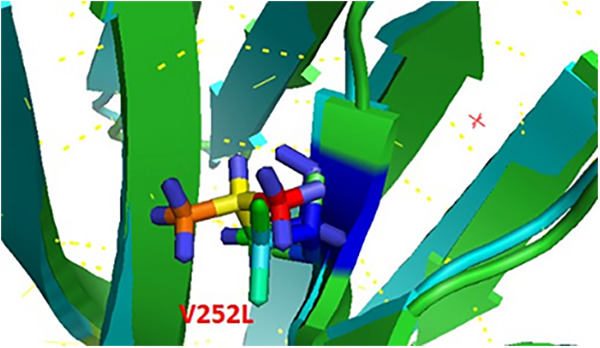
Stereo presentation of HA (hemagglutinin from A/Shiraz/1/2013) with location of V252L created by PyMOL.

Areas outside the defined antigenic sites on the HA head can also be binding sites for antibodies (30). In many cases, these outside regions are more conserved and antibodies targeting these regions may provide more protection against influenza virus (31). V252 and Leu252 are located outside the defined antigenic sites, have a similar functional group, and do not seem to cause much structural change (Fig. 5). Free-energy analysis showed that the V252L mutation has a structural destabilizing effect (Table 1). The change V252L mutation eliminates interactions with A181, F147, L151, and Trp153 and forms new interactions with G195, N264, F161, M244, Y246, L165, and W167, which change the stability of the HA structure (Fig. 5 and Table 4).

**TABLE 4:** Characteristics of noncovalent interactions of V252 and L252 with other residues^*a*^

Pos	Chain	Res	Atom	Pos	Chain	Res	Atom	MO	Dd-a	Dh-a	A(d-H-N)
181	A	G	N	252	A	V	O	2.86	1.88	171.80	139.85
147	Phe	A		252	A	Val					
151	Leu	A		252	A	Val					
153	Trp	A		252	A	Val					
195	A	G	N	266	A	L	O	2.96	1.99	165.57	136.20
266	A	L	N	264	A	N	O	3.26	2.94	99.71	103.86
167	Trp	A		266	A	Leu					
161	Phe	A		266	A	Leu					
244	Met	A		266	A	Leu					
246	Tyr	A		266	A	Leu					
165	Leu	A		266	A	Leu					

a Abbreviations: Dd-a, distance between donor and acceptor; Dh-a, distance between hydrogen and acceptor; A(d-H-N), angle between donor-H-N; MO, multiple occupancy; Pos, position; Res, residue.

**FIG 6:**
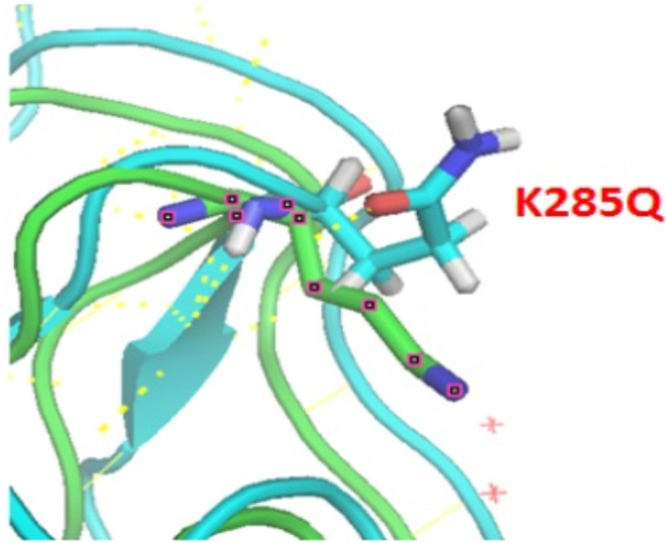
Stereo presentation of HA (hemagglutinin from A/Shiraz/1/2013) with location of K285Q created by PyMOL. Colors show K285 (cyan) and Q285 (lime). We modeled the mutant structure Q285 and superimposed it on the wild-type structure K285.

Mutation K285Q is located in a basic patch in the stalk region of HA (hemagglutinin A/Shiraz/1/2013). Due to the mutation, K285’s interactions with the backbone of I297, T283, and H47 are lost. However, Q285 can interact with H313, T298, and H54 (Fig. 6 and Table 5). This has a destabilizing effect (Table 5).

**TABLE 5:** Characteristics of noncovalent interactions of K285 and Q285 with other residues^*a*^

Pos	Res	Atom	Pos	Res	Atom	Dd-a	Dh-a	A(d-H-N)	A(a-O=C)
285	K	N	283	T	O	2.47	100.91	83.78	
47	H	ND1	285	K	O	3.21	3.15	85.71	115.57
285	K	NZ	297	I	O	2.87	9.99	999.99	150.07
300	Q	N	298	T	O	3.24	81.86	77.47	
300	Q	OE1	313	H	NE2	2.91	2.93	78.90	999.99
54	H	ND1	300	Q	O	3.14	2.94	94.39	100.29

a Abbreviations: Dd-a, distance between donor and acceptor; Dh-a, distance between hydrogen and acceptor; A(d-H-N), angle between donor-H-N; A(a-O=C), angle between acceptor-O=C; MO, multiple occupancy; Pos, position.

**FIG 7:**
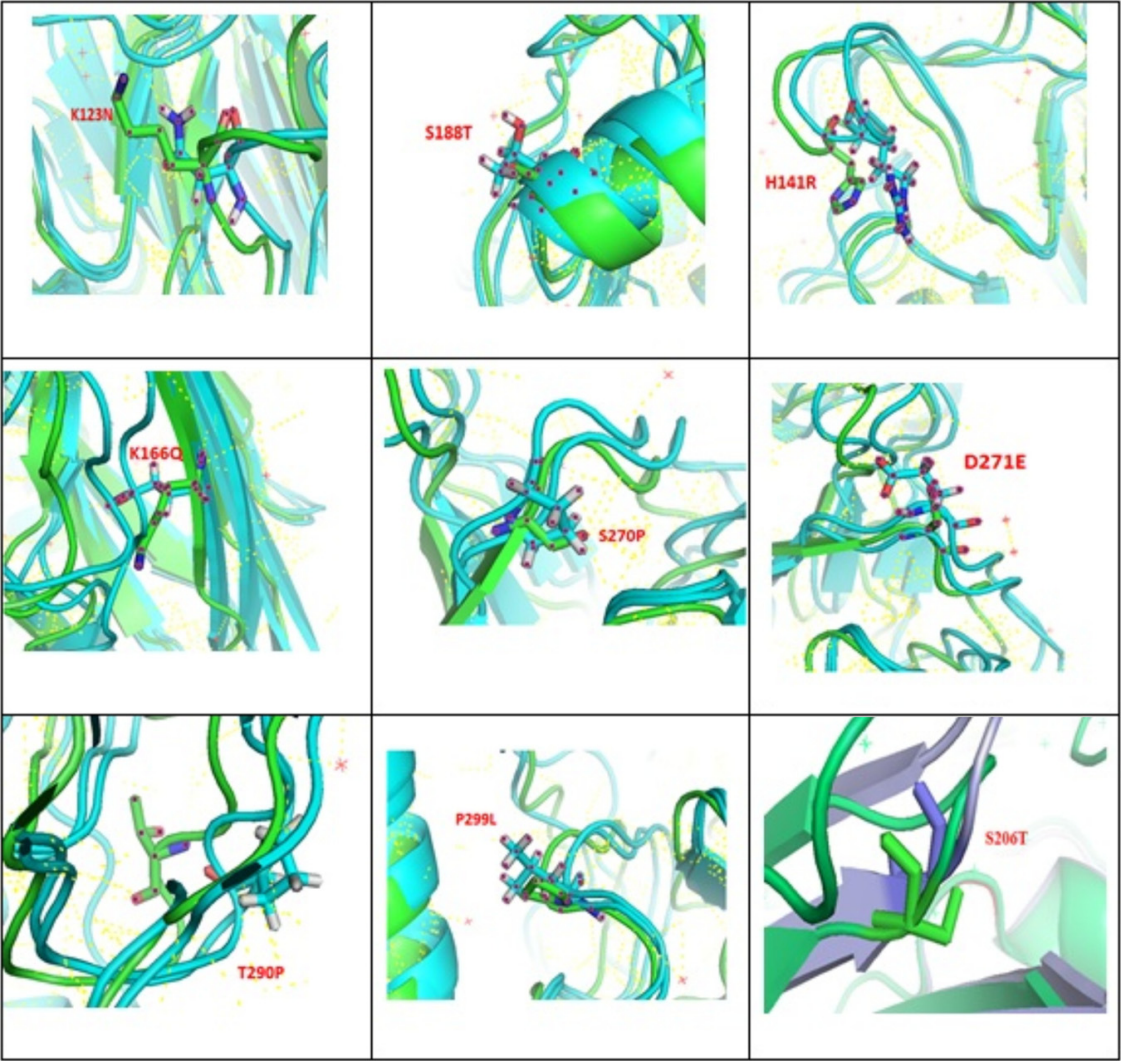
Stereo presentation of HA with locations of K123N, H141R, K166Q, S188T, S206T, S270P, D271E, T290P, and P299L mutations created by PyMOL. Lime shows K123, H141, K166, S188, S206, S270, D271, T290, and P299, and cyan shows N123, R141, Q166, T188, T206, P270, E271, P290, and L299. We modeled the mutant structures and superimposed them on the wild-type structures.

In the wild-type structure of hemagglutinin of A/Shiraz/1/2013, K123, H141, K166, S188, S206, S270, D271, T290, and P299 form intra- and interchain interactions. The following mutations of these residues, K123N, H141R, K166Q, S188T, S206T, S270P, D271E, T290P, and P299L, alter the interactions and cause a destabilizing effect according to the free-energy analysis. The locations of these mutations vary greatly (Fig. 7).

**FIG 8:**
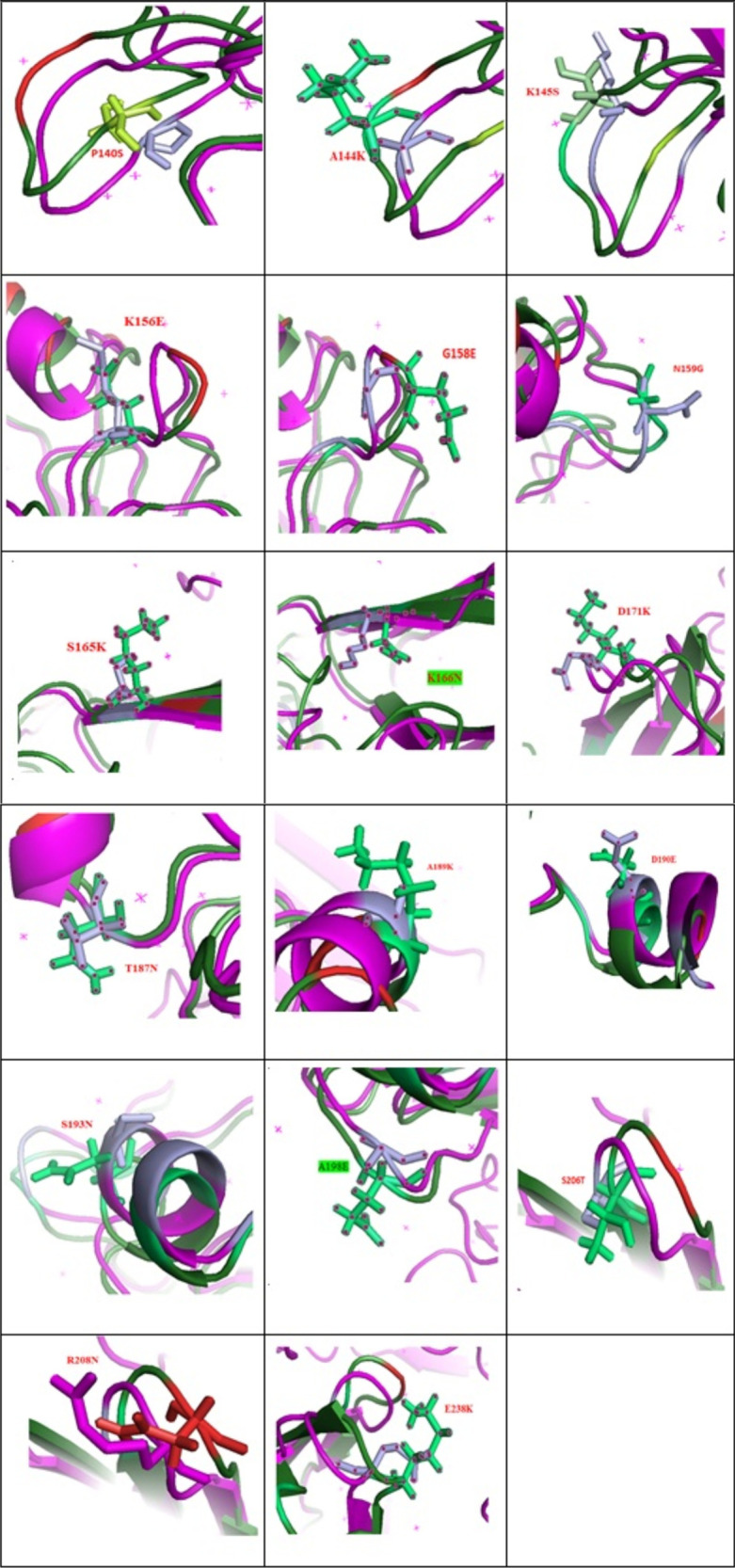
Stereo presentation of HA with locations of P140S, A144K, K145, SK156E, G158E, N159G, S165K, K166N, D171K, T187N, A189K, D190E, S193N, A198E, S206T, R208N, and E238K mutations created by PyMOL. Magenta and light blue show P140, A144, K145, SK156, G158, N159, S165, K166, D171, T187, A189, D190, S193, A198, S206, R208, and E238, and lime and red show S140, K144, S145, E156, E158, G159, K165, N166, K171, N187, K189, E190, N193, E198, T206, N208, and K238. We modeled the mutant structures and superimposed them on the wild-type structures.

There was an overall reduction in stability, as only A198E and K166N mutations increased stability out of the total P140S, A144K, K145, SK156E, G158E, N159G, S165K, K166N, D171K, T187N, A189K, D190E, S193N, A198E, S206T, R208N, and E238K mutations. These mutations are located at different parts of the studied HA (Fig. 8).

This study is a continuation of the previous research that analyzed the molecular diversity of pandemic influenza A(H1N1) viruses in Shiraz (19). As previous studies have shown, the surface proteins of influenza viruses must avoid recognition by the host immune system by modulating their activity and changing the positions of their surface glycoproteins (10). One of the main mechanisms of modulating is antigenic drift, and the influenza A(H1N1)pdm09 virus is an effective model because of the extensive data available for the virus. In this study, we analyzed the structural changes caused by amino acid mutations in the HA of the prevalent influenza A(H1N1)pdm09 virus, which might help the course of influenza treatment.

In the previous study (19), we identified a number of mutations, some of which were previously reported and resulted in antigenic drift. These mutations are located on the antigenic sites Sa, Sb, Ca1, Ca2, and Cb in HA1 and the fusion peptide of HA2. As previously shown, the K166Q mutation is located within the antigenic site Sa (10). This mutation allows the virus to escape from the immune response. The S188T mutation is located in antigenic site Sb and has little impact on the antigenic drift. However, the accumulation of several mutations in different antigenic sites can result in a reduction in vaccine effectiveness.

Previous studies show that in the influenza A(H1N1)pdm09 virus the HA region is unstable (32). This instability leads to various effects on the structure of HA that may benefit the virus (33). In this regard, the mutations, by introducing or disrupting interactions within and between monomers, may have different effects on the stability of the structure of HA. Single point mutations or recruitment of several mutations has different impacts on HA stability.

In the D104N and D472N mutations, deletion of an acidic amino acid masks the acidic patch located in this region. With mutations of K123N, K165Q, and K285Q, the basic patch of this region is obscured. In the N444D and H140R mutations, new acidic and basic patches are formed. In E374K and E499K replacement of an acidic amino acid with an essential one masks the basic patch. Our results show that the alteration of residue interactions at mutation sites causes destabilization of the HA protein. However, this still needs experimental confirmation. An important aspect of influenza virus infection of airway epithelial cells is the interaction between the virus hemagglutinin (HA) protein and the corresponding receptor on the host cell. Although the precise nature of the viral receptor is incompletely defined, influenza viruses target glycosylated oligosaccharides that terminate in a sialic acid (SA) residue. These residues are bound to glycans through α2,3, α2,6, or α2,8 linkage by sialyltransferases that are expressed in a cell- and species-specific manner. Influenza viruses primarily target airway epithelial cells via α2,3- and α2,6-type receptors, but the distribution of these receptors in many species is uncertain and may be a significant factor influencing infection. For example, influenza A viruses isolated from avian species preferentially bind to SA receptors that are linked to galactose by Neu5Acα2,3Gal residues, while human strains preferentially bind the Neu5Acα2,6Gal-terminated sugar chains.

Antibodies are one of the most essential types of therapeutic methods. Predicting the antibody-antigen interaction changes upon mutations (ΔΔ*G* binding) is essential for antibody engineering. On the other hand, identifying hot spots is crucial in the antibody-antigen interaction, because as the hot spots are mutated, the affinity is significantly reduced (34). Kinetic, thermodynamic, and structural analyses of mutants provide essential information about the individual amino acids and elucidate the characteristics of the respective antibody-antigen complexes (35). In the phase of vaccine engineering, obtaining structural, thermodynamic, and kinetic data from mutations is crucial. According to the bioinformatics analyses, the interactions of the mutated amino acids with other amino acids in the structure of HA have changed. This change of interactions can lead to a change in the position of the amino acids that affect the binding of the vaccine to the virus. These structural changes of HA have progressed toward instability based on the bioinformatics analysis of the structure. However, these analyses indicate that current influenza vaccines are likely to have a decreased efficacy in the future. The results of this study can be considered for the effective control of pandemic influenza and the development of a more appropriate vaccine. This study emphasizes the importance of bioinformatics analysis in the designing of drugs or vaccines before production.

## MATERIALS AND METHODS

### Influenza virus sequence data extraction and multiple sequence alignment.

For bioinformatics analysis, the hemagglutinin genes of the influenza A virus from the strains A/Shiraz/106/2015(H1N1) (accession no. ARI70442) and A/Shiraz/1/2013 (accession no. KM013707) were selected with the highest number of mutations. In this regard, full-length amino acid sequences of the mutated HA from influenza virus strains A/Shiraz/106/2015(H1N1), A/Shiraz/1/2013, and A/California/07/2009(H1N1) were obtained from GenBank (http://ncbi.nlm.nih.gov/). The protein reference sequences of similar species were obtained from the NCBI database, and their multiple sequence alignment (MSA) was performed by the ClustalW2 program (https://www.ebi.ac.uk/Tools/msa/clustalo/) and MEGA7 (36).

### Molecular modeling of mutated HA.

For *in silico* studies, the crystal structure of the wild-type HA was obtained from the Protein Data Bank (PDB). Also, the 3D structures of mutated HAs were modeled in the I-TASSER web server (20). On this web server, the 3D models are made based on multiple-threading alignments by LOMETS (37), and we chose the model with the best overall confidence score and Z-score from I-TASSER. The locations of mutations were visualized and studied using the PyMOL Molecular Graphics System (23) and the Swiss PDB Viewer (38). The ExPASy ProtParam server (39) was used to compute the physicochemical parameters of these models such as the molecular weight, the aliphatic index, the total number of positive and negative residues, the extinction coefficient, the theoretical isoelectric point (pI), the instability index, and the grand average hydropathy (GRAVY). The root mean square deviation (RMSD) between the corresponding atoms of two proteins was used to measure the similarity between two protein structures. The template modeling score (TM-score) is a measure of similarity between two protein structures. Hydrogen bonds were also calculated by the WHAT IF web server (25) and the PIC web server (26).

### Study of mutation effect on protein stability.

The effect of these mutations on the stability of mutated HA was evaluated on the iStable server (http://predictor.nchu.edu.tw/iStable/). The iStable server is a web server that predicts the value of free-energy change (ΔΔ*G*) using a support vector machine as an integrator algorithm (40). The iStable server integrates the results of i-Mutant2.0, AUTO-MUTE, MUpro, PoPMuSiC, and CUPSAT programs in the stability prediction and, with the support of a support vector machine, evaluates the point mutation effects on protein stability.

## References

[1] Keilman LJ. 2019. Seasonal influenza (flu). Nurs Clin North Am 54:227–243. doi:10.1016/j.cnur.2019.02.009. 31027663

[2] Pinto AK, Williams GD, Szretter KJ, White JP, Proença-Módena JL, Liu G, Olejnik J, Brien JD, Ebihara H, Mühlberger E, Amarasinghe G, Diamond MS, Boon ACM. 2015. Human and murine IFIT1 proteins do not restrict infection of negative-sense RNA viruses of the *Orthomyxoviridae*, *Bunyaviridae*, and *Filoviridae* families. J Virol 89:9465–9476. doi:10.1128/JVI.00996-15. 26157117 PMC4542382

[3] Cox R, Brokstad K, Ogra P. 2004. Influenza virus: immunity and vaccination strategies. Comparison of the immune response to inactivated and live, attenuated influenza vaccines. Scand J Immunol 59:1–15. doi:10.1111/j.0300-9475.2004.01382.x. 14723616

[4] Cheung TK, Poon L. 2007. Biology of influenza A virus. Ann N Y Acad Sci 1102:1–25. doi:10.1196/annals.1408.001. 17470908

[5] Ilyushina NA, Komatsu TE, Ince WL, Donaldson EF, Lee N, O’Rear JJ, Donnelly RP. 2019. Influenza A virus hemagglutinin mutations associated with use of neuraminidase inhibitors correlate with decreased inhibition by anti-influenza antibodies. Virol J 16:149. doi:10.1186/s12985-019-1258-x. 31783761 PMC6884823

[6] Caton AJ, Brownlee GG, Yewdell JW, Gerhard WJC. 1982. The antigenic structure of the influenza virus A/PR/8/34 hemagglutinin (H1 subtype). Cell 31:417–427. doi:10.1016/0092-8674(82)90135-0. 6186384

[7] Mathews JD, Chesson JM, McCaw JM, McVernon J. 2009. Understanding influenza transmission, immunity and pandemic threats. Influenza Other Respir Viruses 3:143–149. doi:10.1111/j.1750-2659.2009.00089.x. 19627371 PMC4634682

[8] Garten RJ, Davis CT, Russell CA, Shu B, Lindstrom S, Balish A, Sessions WM, Xu X, Skepner E, Deyde V, Okomo-Adhiambo M, Gubareva L, Barnes J, Smith CB, Emery SL, Hillman MJ, Rivailler P, Smagala J, de Graaf M, Burke DF, Fouchier RA, Pappas C, Alpuche-Aranda CM, López-Gatell H, Olivera H, López I, Myers CA, Faix D, Blair PJ, Yu C, Keene KM, Dotson PD, Jr, Boxrud D, Sambol AR, Abid SH, St George K, Bannerman T, Moore AL, Stringer DJ, Blevins P, Demmler-Harrison GJ, Ginsberg M, Kriner P, Waterman S, Smole S, Guevara HF, Belongia EA, Clark PA, Beatrice ST, Donis R, Katz J, Finelli L, Bridges CB, Shaw M, Jernigan DB, Uyeki TM, Smith DJ, Klimov AI, Cox NJ. 2009. Antigenic and genetic characteristics of swine-origin 2009 A (H1N1) influenza viruses circulating in humans. Science 325:197–201. doi:10.1126/science.1176225. 19465683 PMC3250984

[9] Ambroggio XI, Dommer J, Gopalan V, Dunham EJ, Taubenberger JK, Hurt DE. 2013. HASP server: a database and structural visualization platform for comparative models of influenza A hemagglutinin proteins. BMC Bioinformatics 14:197. doi:10.1186/1471-2105-14-197. 23777206 PMC3693987

[10] Castelán-Vega JA, Magaña-Hernández A, Jiménez-Alberto A, Ribas-Aparicio RMJA. 2014. The hemagglutinin of the influenza A(H1N1)pdm09 is mutating towards stability. Adv Appl Bioinform Chem 7:37–44. doi:10.2147/AABC.S68934. 25328411 PMC4198066

[11] Wilson JR, Guo Z, Tzeng W-P, Garten RJ, Xiyan X, Blanchard EG, Blanchfield K, Stevens J, Katz JM, York IA. 2015. Diverse antigenic site targeting of influenza hemagglutinin in the murine antibody recall response to A(H1N1)pdm09 virus. Virology 485:252–262. doi:10.1016/j.virol.2015.08.004. 26318247 PMC5737639

[12] Antón A, Pozo F, Niubó J, Casas I, Pumarola T. 2012. Influenza A(H1N1)pdm09 virus: viral characteristics and genetic evolution. Enferm Infecc Microbiol Clín 30(Suppl 4):10–17. doi:10.1016/S0213-005X(12)70099-X. 23116787

[13] World Health Organization. 2011. Summary of influenza antiviral susceptibility surveillance findings, September 2010–March 2011. World Health Organization, Geneva, Switzerland.

[14] Chan PKS, Lee N, Joynt GM, Choi KW, Cheung JLK, Yeung ACM, Lam P, Wong R, Leung B-W, So H-Y, Lam W-Y, Hui DCS. 2011. Clinical and virological course of infection with haemagglutinin D222G mutant strain of 2009 pandemic influenza A (H1N1) virus. J Clin Virol 50:320–324. doi:10.1016/j.jcv.2011.01.013. 21330192

[15] Hurt AC, Hardie K, Wilson NJ, Deng YM, Osbourn M, Leang SK, Lee RTC, Iannello P, Gehrig N, Shaw R, Wark P, Caldwell N, Givney RC, Xue L, Maurer-Stroh S, Dwyer DE, Wang B, Smith DW, Levy A, Booy R, Dixit R, Merritt T, Kelso A, Dalton C, Durrheim D, Barr IG. 2012. Characteristics of a widespread community cluster of H275Y oseltamivir-resistant A (H1N1) pdm09 influenza in Australia. J Infect Dis 206:148–157. doi:10.1093/infdis/jis337. 22561367 PMC3379839

[16] McKimm-Breschkin JL, Williams J, Barrett S, Jachno K, McDonald M, Mohr PG, Saito T, Tashiro M. 2013. Reduced susceptibility to all neuraminidase inhibitors of influenza H1N1 viruses with haemagglutinin mutations and mutations in non-conserved residues of the neuraminidase. J Antimicrob Chemother 68:2210–2221. doi:10.1093/jac/dkt205. 23759505 PMC3772742

[17] Mir MA, Lal RB, Sullender W, Singh Y, Garten R, Krishnan A, Broor S. 2012. Genetic diversity of HA1 domain of hemagglutinin gene of pandemic influenza H1N1pdm09 viruses in New Delhi, India. J Med Virol 84:386–393. doi:10.1002/jmv.23205. 22246823

[18] Yasugi M, Kubota-Koketsu R, Yamashita A, Kawashita N, Du A, Misaki R, Kuhara M, Boonsathorn N, Fujiyama K, Okuno Y, Nakaya T, Ikuta K. 2013. Emerging antigenic variants at the antigenic site Sb in pandemic A (H1N1) 2009 influenza virus in Japan detected by a human monoclonal antibody. PLoS One 8:e77892. doi:10.1371/journal.pone.0077892. 24147093 PMC3797713

[19] Tavakoli F, Moattari A, Shamsi Shahr Abadi M, Kadivar MR, Khodadad N, Pirbonyeh N, Emami A. 2015. Antigenic variation of the haemagglutinin gene of the influenza A (H1N1) pdm09 virus circulating in Shiraz, February-April 2013. Iran J Immunol 12:198–208. 26412638 10.22034/iji.2015.16749

[20] Zhang Y. 2008. I-TASSER server for protein 3D structure prediction. BMC Bioinformatics 9:40. doi:10.1186/1471-2105-9-40. 18215316 PMC2245901

[21] Wagner R, Matrosovich M, Klenk HD. 2002. Functional balance between haemagglutinin and neuraminidase in influenza virus infections. Rev Med Virol 12:159–166. doi:10.1002/rmv.352. 11987141

[22] Kaplan W, Littlejohn TG. 2001. Swiss-PDB viewer (deep view). Brief Bioinform 2:195–197. doi:10.1093/bib/2.2.195. 11465736

[23] DeLano WL. 2002. The PyMOL molecular graphics system. DeLano Scientific, San Carlos, CA.

[24] Xu R, Ekiert DC, Krause JC, Hai R, Crowe JE, Jr, Wilson IA. 2010. Structural basis of preexisting immunity to the 2009 H1N1 pandemic influenza virus. Science 328:357–360. doi:10.1126/science.1186430. 20339031 PMC2897825

[25] Vriend G. 1990. WHAT IF: a molecular modeling and drug design program. J Mol Graph 8:52–56. doi:10.1016/0263-7855(90)80070-v. 2268628

[26] Tina K, Bhadra R, Srinivasan N. 2007. PIC: protein interactions calculator. Nucleic Acids Res 35:W473–W476. doi:10.1093/nar/gkm423. 17584791 PMC1933215

[27] Tokuriki N, Stricher F, Serrano L, Tawfik DS. 2008. How protein stability and new functions trade off. PLoS Comput Biol 4:e1000002. doi:10.1371/journal.pcbi.1000002. 18463696 PMC2265470

[28] Nachbagauer R, Salaun B, Stadlbauer D, Behzadi MA, Friel D, Rajabhathor A, Choi A, Albrecht RA, Debois M, García-Sastre A, Rouxel RN, Sun W, Palese P, Mallett CP, Innis BL, Krammer F, Claeys C. 2019. Pandemic influenza virus vaccines boost hemagglutinin stalk-specific antibody responses in primed adult and pediatric cohorts. NPJ Vaccines 4:51. doi:10.1038/s41541-019-0147-z. 31839997 PMC6898674

[29] Tan GS, Krammer F, Eggink D, Kongchanagul A, Moran TM, Palese P. 2012. A pan-H1 anti-hemagglutinin monoclonal antibody with potent broad-spectrum efficacy *in vivo*. J Virol 86:6179–6188. doi:10.1128/JVI.00469-12. 22491456 PMC3372189

[30] Matsuzaki Y, Sugawara K, Nakauchi M, Takahashi Y, Onodera T, Tsunetsugu-Yokota Y, Matsumura T, Ato M, Kobayashi K, Shimotai Y, Mizuta K, Hongo S, Tashiro M, Nobusawa E. 2014. Epitope mapping of the hemagglutinin molecule of A/(H1N1)pdm09 influenza virus by using monoclonal antibody escape mutants. J Virol 88:12364–12373. doi:10.1128/JVI.01381-14. 25122788 PMC4248900

[31] Hong M, Lee PS, Hoffman RMB, Zhu X, Krause JC, Laursen NS, Yoon S-I, Song L, Tussey L, Crowe JE, Ward AB, Wilson IA. 2013. Antibody recognition of the pandemic H1N1 influenza virus hemagglutinin receptor binding site. J Virol 87:12471–12480. doi:10.1128/JVI.01388-13. 24027321 PMC3807900

[32] Farnsworth A, Cyr TD, Li C, Wang J, Li X. 2011. Antigenic stability of H1N1 pandemic vaccines correlates with vaccine strain. Vaccine 29:1529–1533. doi:10.1016/j.vaccine.2010.12.120. 21211583

[33] Yang H, Chang JC, Guo Z, Carney PJ, Shore DA, Donis RO, Cox NJ, Villanueva JM, Klimov AI, Stevens J. 2014. Structural stability of influenza A(H1N1)pdm09 virus hemagglutinins. J Virol 88:4828–4838. doi:10.1128/JVI.02278-13. 24522930 PMC3993803

[34] Abbott WM, Damschroder MM, Lowe DC. 2014. Current approaches to fine mapping of antigen–antibody interactions. Immunology 142:526–535. doi:10.1111/imm.12284. 24635566 PMC4107663

[35] Akiba H, Tsumoto K. 2015. Thermodynamics of antibody–antigen interaction revealed by mutation analysis of antibody variable regions. J Biochem 158:1–13. doi:10.1093/jb/mvv049. 25956164

[36] Kumar S, Stecher G, Tamura K. 2016. MEGA7: molecular evolutionary genetics analysis version 7.0 for bigger datasets. Mol Biol Evol 33:1870–1874. doi:10.1093/molbev/msw054. 27004904 PMC8210823

[37] Wu S, Zhang Y. 2007. LOMETS: a local meta-threading-server for protein structure prediction. Nucleic Acids Res 35:3375–3382. doi:10.1093/nar/gkm251. 17478507 PMC1904280

[38] Guex N, Peitsch MC. 1997. SWISS-MODEL and the Swiss-Pdb Viewer: an environment for comparative protein modeling. Electrophoresis 18:2714–2723. doi:10.1002/elps.1150181505. 9504803

[39] Gasteiger E, Hoogland C, Gattiker A, Duvaud S, Wilkins MR, Appel RD, Bairoch A. 2005. Protein identification and analysis tools on the ExPASy server, p 571–607. *In* Walker JM (ed), The proteomics protocols handbook. Humana Press, Totowa, NJ.

[40] Chen C-W, Lin M-H, Liao C-C, Chang H-P, Chu Y-W. 2020. iStable 2.0: predicting protein thermal stability changes by integrating various characteristic modules. Comput Struct Biotechnol J 18:622–630. doi:10.1016/j.csbj.2020.02.021. 32226595 PMC7090336

